# Gastric dilatation volvulus in dogs: utility of lactate as a predictor of survival

**DOI:** 10.18849/ve.v7i4.537

**Published:** 2022-11-02

**Authors:** David Mackenzie+

**Keywords:** CANINE, GASTRIC DILATATION, GASTRIC DILATATION VOLVULUS, GASTRIC TORSION, GDV, LACTATE

## Abstract

PICO question

In dogs presenting with gastric dilatation volvulus, is an admission or pre-operative lactate level a reliable predictor of survival to discharge?

Clinical bottom line

Category of research question

Prognosis

The number and type of study designs reviewed

15 studies (12 retrospective and 3 prospective) were critically appraised

Strength of evidence

Moderate

Outcomes reported

At a population level, lower blood lactate concentration, or lactate concentration that decreases following fluid resuscitation, are associated with a better prognosis.

At a population level, higher blood lactate concentration, or lactate concentration that fails to decrease following fluid resuscitation, is associated with a worse prognosis. However, the lower sensitivity across studies means that a high lactate, or one that does not decrease following fluid therapy, should be interpreted more cautiously than a low lactate; i.e., low lactate predicts survival better than high lactate predicts non-survival.

In all studies, there was a significant overlap in individual blood lactate concentration between survivors and non-survivors

Conclusion

Blood lactate level should only be used to help guide broad, cautiously worded conversations with owners. It should not be used to give a prognosis for individual patients. The overlap between survivors and non-survivors, and the high overall survival rate, suggest that exploratory laparotomy should be advised irrespective of the blood lactate level

How to apply this evidence in practice

The application of evidence into practice should take into account multiple factors, not limited to: individual clinical expertise, patient’s circumstances and owners’ values, country, location or clinic where you work, the individual case in front of you, the availability of therapies and resources.

Knowledge Summaries are a resource to help reinforce or inform decision making. They do not override the responsibility or judgement of the practitioner to do what is best for the animal in their care.

## Clinical scenario

You are presented with a dog in which the clinical and radiographic findings are consistent with a diagnosis of gastric dilatation volvulus (GDV / gastric torsion). You advise initial stabilisation followed by surgical de-rotation and gastropexy as the treatment for this condition. Before embarking on surgery with high associated costs, the owners wish to know the likelihood of survival to discharge. You suggest that measurement of blood lactate concentration may help answer this question.

## The evidence

15 studies (12 retrospective and 3 prospective) were identified and appraised based on the predefined inclusion criteria. Four further studies examining lactate concentration in dogs with a range of conditions (not restricted to GDV), and two review papers, were also appraised.

## Summary of the evidence

**De Papp et al. (1999):** 

Population:	Dogs with gastric dilatation volvulus (GDV) confirmed radiographically or at necropsy. Mixed population represented. Lactate measured using Stat Profile Plus 9, Nova Biomedical.
Sample size:	102 dogs, of which 23 dogs received fluid therapy prior to measurement of lactate concentration.
Intervention details:	One dog died prior to surgery. It is implied that the remaining 101 dogs underwent surgery for GDV.
Study design:	Retrospective observational case-control study./td>
Outcome Studied:	Correlation between initial admission plasma lactate concentration and survival to discharge. Correlation between initial admission plasma lactate concentration and gastric necrosis.
Main Findings (relevant to PICO question):	Overall survival of dogs undergoing surgery was 87/101 (86%). Survival of dogs with lactate <6.0 mmol/L (69/70, [99%]) was significantly higher than dogs with lactate >6.0 mmol/L [18/31, 58%]) (sensitivity = 68%, specificity 88%; p <0.001). Logistic regression analysis showed an increasing probability of survival with decreasing pretreatment lactate level.
Limitations:	Retrospective study. 23 dogs received fluid therapy prior to lactate measurement but these were included in the overall analysis together with dogs that did not receive such treatment.

**Zacher et al. (2010):** 

Population:	Dogs with gastric dilatation volvulus (GDV) confirmed radiographically and at surgery, no pretreatment prior to referral, > 1 measurement of plasma lactate level prior to surgery (pre and post-fluid therapy and stabilisation prior to surgery). Mixed population represented. Lactate measured using Stat Profile 9, Nova Biomedical.
Sample size:	64 dogs.
Intervention details:	All dogs underwent resuscitation with fluid and decompression followed by corrective surgery.
Study design:	Retrospective observational case-control study.
Outcome Studied:	Correlation between admission blood lactate level and survival to discharge, and lactate clearance following resuscitation and survival to discharge.
Main Findings (relevant to PICO question):	Overall survival 49/64 (77%). Initial plasma lactate for survivors (6.2 ± 3.2 mmol/L) was significantly lower than non-survivors (10.3 ± 3.2 mmol/L) p <0.05. Final lactate concentration (post fluids and decompression) for survivors (3.3 ± 2.3 mmol/L) was significantly lower than non-survivors (8.0 ± 3.3 mmol/L) p <0.05. Percentage change in lactate between initial level and the level post fluids and decompression was significantly different between survivors (49.1 ± 28.8%) and non-survivors (24.6 ± 19.4%) p <0.05, although absolute change was not significant. For all dogs, a cut-off for initial lactate of 9.0 mmol/L predicted survival with sensitivity 74% and specificity 73%. A cut-off for final lactate of 5.6 mmol/L predicted survival with sensitivity 84% and specificity 80%. A percentage change in lactate cut-off of 42.2% predicted survival with 61% and specificity 100%. Subset of dogs with 'high lactate' (HIL) were analysed separately. In this group, initial lactate did not differ between survivors and non-survivors. Final lactate (post fluids and decompression) was significantly different between survivors (5.1 ± 2.2 mmol/L) and non-survivors (9.9 ± 2.7 mmol/L) p <0.05. Absolute change in lactate was significantly different between survivors (6.3 ± 2.0 mmol/L) and non-survivors (2.6 ± 2.3 mmol/L) p <0.05. Percentage change in lactate was significantly different between survivors (53.2 ± 17.1%) and non-survivors (18.9 ± 20.5%) p <0.05. For this subset of dogs, a cut-off of 6.4 mmol/L in final lactate (post fluid and decompression) predicted survival with sensitivity 77% and specificity 91%. For this subset of dogs, a lactate cut-off of 4.0 mmol/L in absolute change in lactate predicted survival with sensitivity 92% and specificity 82%. For this subset of dogs, a cut-off of 42.5% in percentage change in lactate predicted survival with sensitivity 85% and specificity 100%.
Limitations:	Retrospective study. Small sample size (power analysis not reported). Three dogs with initial plasma lactate concentration within the reference range were excluded from the study. Variation in pre-surgery fluid therapy. No definitive endpoint for resuscitation was reported. Non-survivors included dogs euthanised at surgery based on subjective impression of gastric necrosis, which may have introduced bias.

**Green et al. (2011):** 

Population:	Dogs with a radiographic diagnosis of gastric dilatation volvulus (GDV), admission blood lactate level measured and subsequent surgery. Mixed population represented. Lactate measured using Stat Profile 9, Nova Biomedical.
Sample size:	84 dogs.
Intervention details:	All dogs underwent stabilisation with intravenous fluid therapy and decompression, followed by corrective surgery for GDV.
Study design:	Retrospective observational case-control study.
Outcome Studied:	Correlation between admission blood lactate level and survival to discharge. Correlation between change in plasma lactate levels (lactate clearance) and survival to discharge. Correlation between admission blood lactate level and presence of gastric wall necrosis. Correlation between gastric wall necrosis and survival to discharge.
Main Findings (relevant to PICO question):	Overall survival 74/84 (88%). Significant difference in admission lactate concentration between non-survivors (median 6.80 mmol/L [range 1.4–16.9 mmol/L]) and survivors (median 3.4 mmol/L [range 0.7–16.1 mmol/L]), p <0.0074. Of 74/84 (88%) that survived, 55/74 (74.4%) of dogs had initial blood lactate <6.0 mmol/L, while 19/74 (25.6%) of dogs had initial blood lactate ≥0 mmol/L. Initial blood lactate <4.1 mmol/L had a sensitivity of 60.3% and specificity 90.9% for predicting survival (P = 0.0133). Serial lactate measurements were available for 52/84 (62%) of dogs. Of these 52 dogs, 40/52 (77%) had a raised (>2.5 mmol/L) initial lactate concentration, and 37/40 (92.5%) of these dogs survived. 26/37 (70%) of these survivors had a reduction in serial lactate of >= 50% from baseline. Of the3/40 (7.5%) of dogs that did not survive, the serial lactate failed to fall ≥ 50% from baseline.
Limitations:	Retrospective study. Some dogs had treatment prior to referral and were not excluded, which may have introduced bias. According to the protocol described in the paper, the treatment that each dog received may have been variable (e.g., some dogs may have received transfusion). Serial lactate results were difficult to interpret, with variability in timing of serial lactate measurements. Also, not clear if some serial lactate results were pre-surgery or post-surgery. Statistical analysis was also not performed on this, including no multivariate analysis, and this may be because the group size was too small. The statistics presented do not readily support the assertion that reduction in lactate from baseline may be a better prognostic indicator than single baseline lactate measurement.

**Green et al. (2012):** 

Population:	Dogs with a radiographic diagnosis of gastric dilatation volvulus (GDV) with measurement of blood lactate level at presentation. Mixed population represented. Lactate measurement method not specified.
Sample size:	101 dogs.
Intervention details:	Dogs underwent corrective surgery for GDV. Other resuscitative interventions not specified.
Study design:	Retrospective observational case-control study.
Outcome Studied:	Survival to discharge correlated with plasma lactate level. Correlation between survival and risk factors for survival (signalment, time of presentation, presenting clinical signs), decompression, and thoracic radiographic findings.
Main Findings (relevant to PICO question):	Overall survival rate 85/101 (84%). Evidence for cardiomegaly on preoperative thoracic radiographs was associated with decreased survival. After controlling for cardiomegaly, lactate <6 mmol/L at presentation had an odds ratio of 7.3 of survival compared with dogs with lactate ≥ 6.
Limitations:	Retrospective study. Lactate cut-off results were reported after controlling for the presence of cardiomegaly. Case selection made on basis of presence of thoracic radiographs which was incomplete, which may have introduced bias. Not clear from the data why a lactate cut-off of 6 mmol/L was taken for calculating odds ratio. Possibilities of Type I and Type II errors due to incomplete records.

**Israeli et al. (2012):** 

Population:	Dogs with a radiographic diagnosis of gastric dilatation volvulus (GDV), confirmed at surgery, and admission blood lactate level measured. Mixed population represented. Lactate measured using either Advia 120 (Siemens Medical) or Abacus or Arcus (Diatron).
Sample size:	66 dogs.
Intervention details:	All dogs underwent corrective surgery for GDV.
Study design:	Blood samples collected prospectively, and records retrospectively reviewed.
Outcome Studied:	Correlation between plasma lactate concentration at admission and survival to discharge. Correlation between plasma lactate concentration at admission and degree of gastric wall necrosis. Correlation between plasma markers serum canine pepsinogen-A, CRP, and cPLI and survival to discharge and degree of gastric wall necrosis.
Main Findings (relevant to PICO question):	Overall survival 51/66 (77.3%). No significant difference in lactate concentration between non-survivors (7.9 mmol/L [range 1.1–25.3 mmol/L] and survivors (4.8 mmol/L [range 0.1–19.1 mmol/L]).
Limitations:	Retrospective study. Non-survivors included dogs euthanised for a variety of reasons including subjective impression of gastric necrosis, owner request, or deterioration during hospitalisation. Variation in resuscitative intervention between dogs not specified. Lactate concentration was analysed using two different analysers. Power analysis not reported.

**Santoro Beer et al. (2013):** 

Population:	Dogs with a radiographic diagnosis of gastric dilatation volvulus (GDV) with no concurrent disease with no prior intervention, and venous blood sample at time of admission, and subsequently underwent corrective surgery for GDV. Mixed population represented. Lactate measured using Stat Profile Critical Care Xpress, Nova Biomedical.
Sample size:	78 dogs.
Intervention details:	68 dogs underwent corrective surgery for GDV (10 dogs were euthanised prior to surgery).
Study design:	Retrospective observational case-control study.
Outcome Studied:	Correlation between admission blood lactate level and survival to discharge. Correlation between presence or absence of gastric necrosis and survival to discharge. Correlation between admission base excess and presence of gastric necrosis and survival to discharge.
Main Findings (relevant to PICO question):	Overall survival rate 65/78 (83%). Initial plasma lactate was significantly associated with survival (non-survivors 9 mmol/L [range 5.6–15 mmol/L), survivors 4.5 mmol/L [range 0.8–14.54 mmol/L] P <0.001). A cut-off admission lactate value of 7.4 mmol/L had sensitivity 75% and specificity 89% for predicting survival to discharge.
Limitations:	Retrospective study. Potential variation in treatment based on initial plasma lactate may have introduced bias. Study included patients euthanised for financial or other unknown reasons.

**O'Neill et al. (2017):** 

Population:	Dogs presenting alive or dead with a confirmed or presumptive diagnosis of gastric dilatation volvulus (GDV), with lactate measured for cases presenting alive. Mixed population represented. Lactate measurement method not specified.
Sample size:	492 dogs total, 483 dogs presented alive of which 285/483 (57.9%) underwent surgery.181/492 cases (36.8%) had blood lactate levels measured.
Intervention details:	285/483 (57.9%) of dogs presenting alive underwent corrective surgery.
Study design:	Retrospective observational cross-sectional study.
Outcome Studied:	Survival to discharge vs non-survival correlated with admission (pretreatment) blood lactate level. Population regression modelling to identify risk factors associated with presumptive diagnosis of GDV.
Main Findings (relevant to PICO question):	Overall survival (of dogs undergoing surgery) was 226/285 (79.3%). For dogs arriving alive, blood lactate concentration at presentation was not associated with likelihood of surgery. Dogs with blood lactate concentrations <4 mmol/L had an increased probability of survival to discharge both overall (P <0·001) and among the surgical cases (P <0·001).
Limitations:	Retrospective study. Multi-centre study with variability in case decision-making and surgery. Diagnosis of GDV was presumptive in some cases. Lactate concentration was not complete (36.8% of presenting population) therefore bias relating to missing data may have been present. Missing data in case records may not have been a random effect which may have introduced bias. 198/492 (40.2%) of cases did not undergo surgery (of which 184/198 [92.9%]) were euthanised. Also, of the 285/492 (57.9%) of dogs that did undergo surgery, 59/285 (20.7%) did not survive, of which 37.59 (62.7%) were euthanised. As the decision to euthanise involves a number of factors, this may have introduced bias into the overall survival rate.

**Verschoof et al. (2017):** 

Population:	Dogs with gastric dilatation volvulus (GDV) confirmed radiographically and for which blood samples were available. Mixed population represented. Lactate concentration measured using blood gas analyser cobab b 221 (Roche Diagnostics, Switzerland).
Sample size:	20 dogs.
Intervention details:	All dogs received preoperative stabilisation with intravenous fluid therapy and gastric needle decompression. All dogs subsequently underwent corrective surgery for GDV.
Study design:	Prospective case control study.
Outcome Studied:	Correlation between survival to discharge and initial blood lactate level. Correlation between initial blood levels of coagulation variables (platelet count, prothrombin time, activated partial thromboplastin time, fibrinogen, antithrombin, protein C, protein S, and D-dimers) and inflammatory biomarkers (CRP, WBC count, lymphocyte and neutrophil numbers) and survival to discharge.
Main Findings (relevant to PICO question):	Overall survival 15/20 (65%). Initial plasma lactate was significantly higher in non-survivors (11.8mmol/L [range 7.5–16.2 mmol/L] than overall survivors (6.2mmol/L [range 1.9–9.7 mmol/L]) p <0.01. Plasma lactate on day 1 post surgery was significantly different between non-survivors and survivors (p = 0.0078). (Values not given, but significant overlap between populations displayed graphically). For surviving dogs, plasma lactate decreased significantly between initial lactate, day 1 and day 2 post-surgery (this data was not compared with non-survivors).
Limitations:	Small sample size (power analysis not reported). Two dogs were euthanised during surgery due to subjective impression of severe gastric necrosis. Five dogs were euthanised post-surgery due to sepsis and disseminated intravascular coagulation (DIC).

**Oron et al. (2018):** 

Population:	Dogs with gastric dilatation volvulus (GDV) that underwent corrective surgery and which had blood samples taken from both cephalic and saphenous veins. Mixed population represented. Lactate measured using a 'dry' analyser or 'wet' chemistry autoanalyser (Reflotron®, Boehringer Mannheim or Reflovet Plus, Roche).
Sample size:	41 dogs in final analysis.
Intervention details:	Fluid resuscitation and gastric decompression (details varied between subjects and between centres). All dogs underwent corrective surgery.
Study design:	Prospective study.
Outcome Studied:	Survival to discharge vs non-survival correlated with admission (pretreatment) blood lactate concentration. Comparison of lactate concentration between cephalic and saphenous sampling sites.
Main Findings (relevant to PICO question):	Overall survival 37/45 (82.9%). No significant difference detected in lactate between the saphenous and cephalic sampling sites. Initial median saphenous and cephalic lactate concentration for all dogs was significantly higher in non-survivors (3.9 mmol/L [range 0.7–17.8 mmol/L] and 3.8 mmol/L [range 0.78–21.9 mmol/L] from saphenous and cephalic sites respectively), compared to survivors (11.2 mmol/L [range 6.6–11.8 mmol/L] and 11.4 mmol/L [range 3.8–19.4 mmol/L] respectively), p = 0.01. When dogs that had undergone gastric decompression prior to blood sampling (n = 10) were removed from the above analysis results remained significant at: non-survivors 10.7mmol/L [range 6.8–11.2 mmol/L] and 11.1mmol/L [range 3.8–11.4 mmol/L] and survivors 3.5 mmol/L [range 0.7–17.8 mmol/L] and 3.1mmol/L [range 0.7–19 mmol/L] (from saphenous and cephalic respectively) p <0.001.
Limitations:	Dogs that died or were euthanised before surgery were excluded from analysis which potentially biases the results by restricting analysis to a more stable population. The total study population was drawn from different populations, a) from a teaching hospital (n = 39) and b) from three specialty referral clinics (n = 6), these populations were combined in the subsequent analysis despite potential variation in resuscitation protocols and treatment. Lactate was analysed using different analysers. Reporting of results for all dogs included those that had already undergone an intervention (gastric decompression).

**Spinella et al. (2018):** 

Population:	Dogs diagnosed with gastric dilatation volvulus (GDV) and dogs diagnosed with intestinal obstruction (IO) (Control group). Mixed population represented. Lactate measured with portable lactate analyser (Lactate Scout +, EKF Diagnostics, Cardiff, UK).
Sample size:	22 dogs with GDV, 16 dogs with IO.
Intervention details:	All dogs in both groups underwent corrective surgery.
Study design:	Prospective observational case-control study.
Outcome Studied:	Survival to discharge vs non-survival correlated with admission (pretreatment) blood lactate level. Correlation between initial admission serum lipase activity, canine pancreatic lipase immunoreactivity (cPLI) and C-reactive protein concentration and survival.
Main Findings (relevant to PICO question):	Overall survival 16/22 (73%). No significant difference between lactate level of non-survivors (7.1 mmol/L [range 3.1–12.1 mmol/L]) and survivors (5.25 mmol/L [range 1.8–10.3 mmol/L]).
Limitations:	Basis for diagnosis of GDV not specified. Control group used was dogs with IO which is not a 'disease free' control group. Presurgical interventions mentioned but not specified and not clear if applied to all dogs. Some dogs (number not stated) were euthanised based on subjective impression of gastric wall necrosis, with potential for bias. Small number of dogs in study and authors state that number of dogs studied was lower than required to make assessment of mortality risk.

**Troia et al. (2018):** 

Population:	Dogs with a clinical and radiographic diagnosis of gastric dilatation volvulus (GDV). Mixed population represented. Lactate measurement technique not specified.
Sample size:	29 dogs.
Intervention details:	Preoperative stabilisation (details not specified). Corrective surgery for GDV.
Study design:	Prospective observational case control study.
Outcome Studied:	Survival to discharge vs non-survival correlated with admission (pretreatment) blood lactate level. Correlation between admission (pretreatment) levels of the biomarkers cell-free DNA, high mobility group box-1, procalcitonin, lactate, and APPLE_fast_ score, with survival, evidence of gastric necrosis, and occurrence of postoperative complications.
Main Findings (relevant to PICO question):	Lactate concentration was not different between survivors and non-survivors.
Limitations:	Three dogs received treatment prior to presentation. Small sample and no power analysis performed**.** Four dogs were euthanised based on subjective assessment of gastric necrosis which may have biased the outcome results. Although blood samples were taken prospectively, records were analysed retrospectively with missing data or variation between subjects (e.g., timing between onset of clinical signs and admission / surgery). Two different centres contributed to the study with potential for differences in case management which may have affected outcomes.

**Grassato et al. (2020):** 

Population:	Dogs with a radiographic or surgical diagnosis of gastric dilatation volvulus (GDV) with no prior treatment, that subsequently underwent corrective surgery, and for which blood lactate measurement was available. Mixed population represented. Lactate measured on presentation (T0) and 24 hours (T24) and 48 hours (T48) after surgery. Method of lactate measurement not specified.
Sample size:	45 dogs.
Intervention details:	All dogs received intravenous fluid resuscitation, antibiotic therapy and gastric decompression via orogastric tube and / or percutaneous needle. All dogs subsequently underwent corrective surgery for GDV.
Study design:	Retrospective observational case-control study.
Outcome Studied:	Survival to discharge vs non-survival correlated with T0, T24 and T48 blood lactate concentration and change in lactate concentration between time points. Presence or absence of gastric necrosis correlated with T0, T24 and T48 blood lactate concentration, and change in lactate concentration between time points.
Main Findings (relevant to PICO question):	Overall survival 31/45 (69%). No significant difference detected in lactate concentration between at any of the three time points (all values in mmol/L). T0: Non-S 3.25 mmol/L (2.1–12.7 mmol/L), S 3.2 mmol/L (0.9–16.4 mmol/L); T24: Non-S 1.1 mmol/L (0.7–9.1 mmol/L), S 1.2 mmol/L (0.6–4.2 mmol/L); T48: Non-S NA (0.6–6.2 mmol/L), S 0.9 mmol/L (0.6–2.5 mmol/L). No significant difference in lactate concentration between either group (survivors versus non-survivors) at any time point. Despite the above, a lactate level of < 4.45 mmol/L was reported as distinguishing survivors from non-survivors with sensitivity 33% and specificity 77%. Lactate concentration for survivors (when reported as mean ± SD in table format) at T24 and T48 was significantly lower than at T0 whereas this was not the case for non-survivors (significance not given).
Limitations:	Retrospective study. Method of lactate measurement not reported, nor how accurate timing of postoperative blood lactate levels was achieved. Small number of cases (power analysis not reported). Data regarding pre-hospitalisation factors was not available. The tabulated data showed survivors had a significant reduction in lactate between time-points compared to non-survivors when data was presented as mean ±SD, but not when reported as median with range. However, reductions were also reported in lactate levels in dogs both with and without gastric necrosis when presented as mean ±SD but not when reported as median with range. This led to a confusing discussion where the authors mixed discussion of gastric necrosis and outcome and concluded that reduction in lactate levels could not be used as a prognostic indicator. Apparent contradiction between results section and table as described above. Significance levels not reported – as described above.

**Rauserova-Lexmaulova et al. (2020):** 

Population:	Dogs with a radiographic diagnosis of gastric dilatation volvulus (GDV) with no prior treatment, that subsequently underwent corrective surgery, with blood lactate samples taken at admission prior to any treatment. Mixed population represented. Initial (pretreatment) lactate measured only. Lactate measured using Konelab 20i analyser (Thermo Fisher Scientific, Finland) processed within 15 minutes or stored frozen and analysed the following day with same analyser.
Sample size:	75 dogs.
Intervention details:	All subjects underwent corrective surgery for GDV. Other interventions not specified.
Study design:	Retrospective observational case-control study.
Outcome Studied:	Survival to discharge vs non-survival correlated with admission (pretreatment) blood lactate level. Presence or absence of gastric necrosis correlated with admission (pretreatment) blood lactate level. Correlation between initial values of pH, PCO_2_, bicarbonate, base excess (BE) and electrolytes (sodium, potassium, chloride, total calcium and inorganic phosphorous), anion gap (AG), strong ion difference (SID) and surgical finding of gastric necrosis and survival to discharge.
Main Findings (relevant to PICO question):	Overall survival 61/75 (81.3%.) Initial plasma lactate concentration was significantly associated with mortality (non-survivors median 9.68 mmol/L, [range 5.84–13.49 mmol/L] vs. survivors, median 3.36 mmol/L, [range 2.42–5.64 mmol/L] p ˂ 0.0001). However, in multivariate logistic regression analysis, lactate concentration was not independently associated with mortality. Optimal cut-off points to predict patient survival were lactate ≤ 5.62 mmol/L (75.4% sensitivity, 85.7% specificity).
Limitations:	Retrospective study. Samples from both arterial and venous sources were used for the comparison of lactate. Pre-surgical intervention / stabilisation was not specified. Risk of possible concurrent diseases (e.g., cardiac, liver, and renal) could not be excluded. Relatively small number of patients (power analysis not undertaken).

**Sharp et al. (2020):** 

Population:	Dogs with a radiographic diagnosis of gastric dilatation volvulus (GDV) with no prior treatment. Mixed population represented. Method of lactate measurement not specified.
Sample size:	498 dogs, of which 429 dogs had lactate measured at presentation.
Intervention details:	116 dogs were euthanised preoperatively, with 382 dogs undergoing corrective surgery.
Study design:	Retrospective observational case-control study.
Outcome Studied:	Survival to discharge vs non-survival correlated with presentation lactate measurement. Non-survivors were further categorised as: euthanised preoperatively, euthanised intraoperatively, euthanised postoperatively, and died.
Main Findings (relevant to PICO question):	Overall survival 275/429 (64.1%). Initial plasma lactate (3.7 mmol/L [range 0.5–20 mmol/L]) was significantly different between survivors and those that were euthanised preoperatively (6.1 mmol/L [range 1.5–19.4 mmol/L]), intra-operatively (8.15 mmol/L [range 2.7–15.7 mmol/L]), and died postoperatively (10.1 mmol/L [1.6–19 mmol/L]) (p <0.001 for all), but not for those that were euthanised postoperatively (5.65 mmol/L [range 2.1–16.5 mmol/L]). Many dogs in the survivor group had very high lactate concentration (range 0.5–20 mmol/L).
Limitations:	Retrospective study. Number of dogs in some outcome groups were quite small (power analysis not reported). Some cases were excluded based on presumptive diagnosis of GDV. High percentage of dogs were euthanised preoperatively (without intent to treat and without radiographic confirmation of diagnosis) which accounted for the majority of mortality in this study, with potential for bias of results. Method of lactate measurement not specified.

**White et al. (2021):** 

Population:	Dogs with a physical and radiographic diagnosis of gastric dilatation volvulus (GDV) and in which orogastric decompression and gastric lavage had been performed. Mixed population represented. Lactate measurement technique not specified.
Sample size:	41 dogs.
Intervention details:	Intravenous fluid resuscitation, gastric decompression, gastric lavage. Surgical correction of GDV (immediate or as a staged procedure as described below).
Study design:	Retrospective observational case-control study.
Outcome Studied:	Survival vs non-survival correlated with initial plasma lactate concentration. Survival vs non-survival correlated with percentage change in plasma lactate concentration following gastric decompression and stabilisation. Survival vs non-survival correlated with performing surgical correction for GDV under the same anaesthesia as for decompression, or as a staged procedure with corrective surgery being performed some hours later following gastric decompression and medical stabilisation prior to exploratory laparotomy.
Main Findings (relevant to PICO question):	Non-survivors had a mean initial plasma lactate concentration of 7.3 mmol/L (range 2.7–11.1 mmol/L) while survivors had a mean initial plasma lactate concentration of 3.2 mmol/L (range 1.2–7.8 mmol/L) (p <0.001). Larger percentage decreases in plasma lactate concentration following decompression and stabilisation were associated with higher likelihood of survival to discharge (p <0.03) – full data not provided but 14/15 dogs that survived decreased their plasma lactate by ≥ 50%.
Limitations:	Retrospective study. Dogs were excluded if orogastric intubation was not included as part of gastric decompression. Dogs were excluded if, following decompression and stabilisation, they were discharged or transferred to another hospital for corrective surgery. Timing of lactate measurement post stabilisation was not standardised.

## Appraisal, application and reflection

Of the 15 studies appraised, many did not have the PICO question given above (association between plasma lactate concentration and outcome) as their primary objective. Rather, plasma lactate was measured as part of a broader study assessing other variables and / or outcomes. Whilst this did not affect results, it meant that, in some cases, statistical analysis was not as detailed or as focused on the PICO question of this Knowledge Summary as might otherwise have been the case (for example, some studies reported receiver operating characteristic (ROC) analysis and optimal sensitivity and specificity, while others did not). This explains why these studies did not have 'lactate' as a title keyword. However, the six studies of Rauserova-Lexmaulovaa et al. (2020), Grassato et al. (2020), Santoro Beer et al. (2013), Green et al. (2011), Zacher et al. (2010) and De Papp et al. (1999) did have the PICO question of the Knowledge Summary (association between survival and plasma lactate level) as the primary study focus.

Twelve of the 15 studies were retrospective, with the limitations inherent to retrospective studies, as described above, in particular there was variation both within and / or between studies in the presenting signs, characteristics of the study population, and analysis and presentation of data.

Median overall survival across all studies = 78.4% (range 64.1–88%).

### Single pre-intervention plasma lactate measurements

Thirteen studies reported an initial (pre-intervention) lactate level in survivors vs non-survivors. In 11 papers this was reported as median with range for the two groups, in one paper (White et al., 2021) this was reported as mean with range for the two groups; while in three studies (O'Neill et al., 2017 Green et al., 2012; and De Papp et al., 1999) this was reported as number of cases above and below a pre-selected lactate cut-off value (> 4 mmol/L in the first and > 6 mmol/L in the latter two studies respectively).

Of the total 15 studies appraised 11 papers reported a significant difference in initial lactate concentration between survivors and non-survivors,4 papers reported no significant difference in initial lactate concentration between survivors and non-survivors.

In all studies, the range of lactate values reported for both survivors (range 0.1–20 mmol/L) and non-survivors (range 1.1 - 25.3 mmol/L) was very wide, with, in all cases, significant overlap in values between the two groups (summarised in the table below). In many of the studies, the sample size was small (as indicated above and below), and in no study was power analysis undertaken to detect a significant difference between lactate levels in survivors versus non-survivors. This raises the possibility that those studies reporting non-significance were under-powered to detect significance in plasma lactate levels between survivors and non-survivors.

Even in those studies where a significant difference was found between populations, the broad range of values (and overlap between survivors and non-survivors) has important implications for decision-making in individual animals. In practical terms, although clinicians may (correctly) infer that a 'high lactate’ (HIL) value is more commonly associated with non-survival compared to a 'lower lactate’ level, the range of lactate values within these two groups, and the substantial overlap between them, makes prediction at the level of individual cases difficult. This data is summarised in the table below:

**Table 1:** Summary of studies appraised in the knowledge summary, showing median lactate concentration and range for survivors vs. non-survivors

Study	Number included in study	Overall survival %	Initial median lactate and range (survivors) mmol/L	Initial median lactate and range (non-survivors) mmol/L	Significance (survivors vs non-survivors)
White et al. (2021)	41	88	Mean 3.2 (1.2–7.8)	Mean 7.3 (2.7–11.1)	P <0.001
Rausterova-Lexmaulovaa et al. (2020)	75	81.3	3.36 (2.42–5.64)	9.68(5.84–13.49)	P <0.0001
Grassato et al. (2020)	45	69	3.2 (0.6–16.4)	3.25 (2.1–12.7)	NS
Sharp et al. (2020)	429	64.1	3.7 (0.2–20)	6.1 (1.5–19.4) [cases euthanised pre-operative]; 8.15 (2.7–15.7) [euthanised intra-operative]; 10.1 (1.6–19) [died postoperative]	P <0.001
Oron et al. (2018)	45	82.9	3.9 (0.7–17.8)3.8 (0.78–21.9)*	11.2 (6.6–11.8)*11.4 (3.8–19.4)**	P = 0.01
Spinella et al. (2018)	22	73	5.25 (1.8–10.30)	7.1 (3.1–12.1)	NS
Troia et al. (2018)	29	76	2.8 (1.1–10.6)	5.2 (2.6–11.9)	NS
O’Neill et al. (2017)	181	79.3	Stratified ≤4 associated with increased survival	–	P <0.001
Verschoof et al. (2017)	20	75	6.2 (1.9–9.7)	11.8 (7.5–16.2)	P <0.01
Santoro Beer et al. (2013)	78	83	4.5 (0.8–14.4)	7.9 (5.6–15)	P <0.001
Green et al. (2011)	84	88	3.4 (0.7–16.1)	6.80 (1.4–16.9)	P <0.0074
Green et al. (2012)	101	84	–	–	–
Israeli et al. (2012)	66	77.3	4.8 (0.1–19.9)	7.9 (1.1–25.3)	NS
Zacher et al. (2010)	64	77	6.2 (0–12.6)	10.3 (3.9–16.7)	P <0.05
De Papp et al. (1999)	102	Lactate <6 mmol/L 99%; >6 mmol/L 58%	3.5 (0.1–14.1)	8.5 (2–13.8)	P <0.001
*/**Concentration reported measured from saphenous* and cephalic** vein respectively					

## Receiver operating characteristic (ROC) analysis

Six studies performed receiver operating characteristic (ROC) analyses to determine a cut-off for lactate which optimised sensitivity and specificity for survival vs non-survival. The paper of De Papp et al. (1999) stated that a cut-off lactate value of 6 mmol/L was chosen so as to slightly increase specificity at the expense of sensitivity, while in the other studies it was implied that a cut-off which maximised both sensitivity and specificity was chosen. However, this data is problematic for clinicians faced with making decisions for individual patients, because the selected optimal cut-offs across studies ranged from a lactate of 4.1 to 9.0 mmol/L. In other words, it is not clear from the literature what lactate value should be used as a cut-off to optimise sensitivity and specificity. In all studies, the sensitivity was moderately low (highest 75.4%, range 60.3–75.4%) while the specificity was somewhat higher (highest 90.9%, range 73–90.9%). The paper of Green et al. (2012) expressed the cut-off as an odds ratio of survival, rather than sensitivity and specificity. This data is summarised in the table below:

**Table 2:** Summary of studies in which Receiver Operating Curve (ROC) analysis was reported, showing optimum cut-off in lactate concentration for survival vs. non-survival and associated sensitivity and specificity

Study	Plasma lactate cut-off for survival to discharge (mmol/L)	Sensitivity %	Specificity %
Green et al. (2011)	< 4.1	60.3	90.9
Rauserova-Lexmaulovaa et al. (2020)	≤ 5.62	75.4	85.7
Green et al. (2012)	< 6	Odds ratio 7.3	–
De Papp et al. (1999)	< 6	61	88
Santoro Beer et al. (2013)	< 7.4	75.0	89.0
Zacher et al. (2010)	< 9.0	74	73

The problem of a low sensitivity is the relatively high false negative rate therefore, a significant proportion of dogs would be classified falsely as having a 'low lactate' (i.e., below the cut-off and therefore considered more likely to survive). The implication of this is that such dogs (approximately 25% with a sensitivity of 75%) may be given a falsely optimistic prognosis. In practical terms this may affect the decision to proceed to surgery (with cost implications for the owner) but with a poor outcome.

This is, arguably, less problematic than having a low specificity, with the corresponding false positive rate. For example, taking a specificity of 88%, would mean 12% of dogs being given a falsely poor prognosis. Such dogs may, in fact, have a good outcome but the owners may be advised otherwise (i.e., advised not to proceed with surgery and instead consider euthanasia) in this proportion of cases.

However, having these statistics allows the clinician to advise owners accordingly, for example a dog with a 'high lactate' (i.e., above the chosen cut-off) has an approximate 75% chance that this is a true value and that the prognosis for survival to discharge should be correspondingly guarded. Conversely, a dog with a 'low lactate' (i.e., below the chosen cut-off) has an approximately 86% chance that this is a true value and a more optimistic outlook for survival to discharge should be given. The summary of these findings is that a 'low' plasma lactate concentration is, in general, a better predictor of survival than a 'high' plasma lactate concentration is a predictor of non-survival, and owners should be advised accordingly. The principal difficulty is in choosing the most appropriate cut-off, given the variation in this value in the literature.

The logistic regression analysis provided in the paper of De Papp et al. (1999) provides, perhaps, the most useful representation of data, and goes some way to addressing this question, as it gives a graphical representation of probability of survival vs plasma lactate concentration. In this paper, a preoperative plasma lactate of 6 mmol/L gave an approximate survival to discharge of 90%. This probability then fell steeply and almost linearly for lactate values > 6 mmol/L, with a lactate level of 8 mmol/L associated with an approximate 70% survival, and 10 mmol/L with an approximate 50% survival (De Papp et al., 1999).

### Change in lactate concentration following intervention (lactate clearance)

In addition to assessing lactate concentration at a static point in time, five papers examined changes in lactate concentration (lactate clearance) over a determined period of time. These findings are summarised in the table below:

**Table 3:** Summary of studies in which change in lactate concentration over time (lactate clearance) was reported, and its relationship to survival

Study	Difference in initial lactate survivors vs non-survivors	Time points	Results
White et al. (2021)	Yes p <0.001	Admission, then at variable time points following fluid stabilisation and decompression	The paper reports that larger percentage decreases in lactate concentration (following stabilisation and decompression) were associated with high likelihood of survival to discharge (p = 0.03), further statistics were not provided but 14/15 dogs that survived decreased their plasma lactate by ≥ 50%.
Grassato et al. (2020)	No	Admission (T0), then 24hrs (T24) and 48hrs (T48) post-surgery	No significant difference between survivors and non-survivors at any time point. The results section suggested that surviving dogs reduced their lactate concentration by a greater amount than non-survivors, however this appeared to be contradicted in the discussion section (see comments below). Significance levels were not reported.
Zacher et al. (2010)	Yes, p <0.05	Initial (admission) and then following fluids and gastric decompression.	Final lactate concentration (post fluids and decompression) for survivors (3.3 ± 2.3) was significantly lower than non-survivors (8.0 ± 3.3) p <0.05. Percentage change in lactate between initial level and the level post fluids and decompression was significantly different between survivors (49.1 ± 28.8%) and non-survivors (24.6 ± 19.4%) p <0.05, although absolute change was not significant. A percentage change in lactate cut-off of 42.2% predicted survival with sensitivity 61% and specificity 100%.
Verschoof et al. (2017)	Yes, p <0.0078	Admission, then day 1 post-surgery.	Lactate concentration day 1 after surgery was significantly different between survivors and non-survivors.
Green et al. (2011)	Yes, p <0.0074	Admission, then variable time-points, median 6hrs later.	70% of surviving dogs showed a reduction in lactate of >50% from baseline.

The study of Grassato et al. (2020) has limitations as significance levels were not given, and some statements in the text of the results and discussion section appeared to be contradictory to those of the tabulated data. For example, significant differences were described in the results table between mean initial lactate concentration (T0) and concentration at both T24 and T48 for dogs without gastric necrosis and dogs surviving; and at T24 only for dogs with gastric necrosis. No differences in any group and at any time point were recorded in the results table for median values. However, the terms mean and median were interchanged in the text of the discussion section. The discussion also stated that a decrease in median lactate of ≥ 50% was detected for 'all the considered categories' however no significant differences were recorded in the results table, so it was not clear which description was correct. The absence of stated significance levels, and the discussion of gastric necrosis and survival together (without multivariate analysis) made the results and discussion difficult to understand. The four other papers all reported that lactate concentration reduced to a greater extent in survivors compared to non-survivors (as well as showing a difference in static initial lactate concentration). It is difficult to directly compare these studies, as the time points for subsequent lactate measurements were different, following fluid plus decompression in Zacher at al. (2010), at variable time-points in Green et al. (2011) and White et al. (2021) and pre- and post-surgery in Verschoof et al. (2017).

The most challenging initial decision facing owners of a dog presenting for GDV is whether to proceed to surgery or not, and risk incurring charges of (usually) several thousand pounds. Guidance on likely outcome pre-surgery would be the most useful. The paper of Zacher et al. (2010) may be the most helpful in guiding practitioners since it looked at lactate pre- and post-resuscitation but prior to surgery and showed a more significant reduction in lactate in survivors compared to non-survivors. Using a cut-off of change in lactate of 42.2% predicted survival with sensitivity 61% and specificity 100%. In other words, these values suggest a false negative rate of 39% (i.e., 39% of dogs did not reduce their lactate by 42.2% but were survivors); and a false positive rate of 0% (i.e., 0% of dogs reduced their lactate by 42.2% but were not survivors). This suggests that a dog displaying a reduction in lactate of 42.2% should be given a good prognosis, whilst failing to show this reduction in lactate should be interpreted more cautiously. However, when considering this study, the limitations described above should be noted.

The general summary of these papers is that, at a population level, dogs that reduce their lactate concentration compared to admission level are likely to have a better prognosis than those dogs who do not. The difficulty, as for static levels, is in using this information to make decisions for individual animals.

## Additional information from non-GDV studies looking at lactate as a prognostic marker

A number of studies have examined lactate as a prognostic marker in cases other than dogs with GDV. The paper of Stevenson et al. (2007), looking at lactate in a variety of systemically ill dogs (n = 80), concluded that there was no significant difference in initial lactate between survivors and non-survivors; however, a reduction in lactate by ≥ 50% at 6 hours following resuscitation was associated with increased survival.

In the large (n = 566) retrospective study of Kohen et al. (2018), also looking at dogs presenting with a variety of different conditions, admission lactate was found to be a significant independent predictor of survival. Further, univariate analysis showed that odds of mortality increased with an increasing magnitude of hyperlactataemia. However, consistent with the GDV-specific studies, there was a significant overlap in lactate values between survivors and non-survivors.

In the more recent paper of Blutinger et al. (2021), a prospective study looking at lactate in dogs (n = 71) with shock due to a variety of conditions, it was found that there was no difference in mean admission lactate between survivors and non-survivors; however, the percentage change in lactate post-resuscitation was a significant predictor of survival.

Similarly, the recent paper of Ortolani & Bellis (2021), looking at critically ill dogs with a range of different conditions (n = 267), also showed that there was a significant difference in admission lactate concentration between survivors and non-survivors. Again, however, there was a significant overlap in lactate values between survivors and non-survivors.

Power analysis was not reported in any of the above papers. However, the extent of overlap in lactate values between survivors and non-survivors, which is a consistent finding in all of the above studies, suggests that, in those cases with smaller populations, the study was more likely to be under-powered to detect a significant difference in lactate concentration between survivors and non-survivors.

## Summary and application

The overall weight of evidence suggests that, at a population level, admission plasma lactate concentration is significantly different between survivors and non-survivors. However, in all studies, the range of lactate values between survivors (range 0.1–20 mmol/L across all studies) and non-survivors (range 1.1–19.4 mmol/L across all studies) was very broad, with significant overlap in values between the two populations. This likely reflects, firstly, that many factors contribute to elevations in lactate, and secondly, that the magnitude of increase in plasma lactate does not indicate its reversibility. The measured plasma lactate concentration at any given time is the balance between tissue production vs metabolism and excretion. In dogs with GDV, the main causes of lactate production are proposed to be from gastric necrosis, and systemic distributive shock, and gastric necrosis is believed to be the main factor affecting survival (De Papp et al., 1999; Zacher et al., 2010; Green et al., 2011; Santoro Beer et al., 2013; and Mooney et al., 2014). As treatment protocols differed between the studies described, direct comparison between studies, and in particular determination of a specific lactate level that should be used to guide decision-making, is difficult.

The differences in sensitivity and specificity discussed above, also suggest that a high lactate should be interpreted with somewhat greater caution when advising owners compared with a low lactate. In general, a dog with a lower lactate, or one which shows a reduction in lactate following fluid resuscitation, should be given a better prognosis, and corrective surgery would be recommended. A dog with a higher lactate, or one which does not decrease following adequate fluid resuscitation, should cautiously be given a more guarded prognosis, but owners should be advised that many dogs with higher lactate, and those that do not have a decrease in lactate following fluid resuscitation, may still have a reasonable expectation of survival, and exploratory laparotomy should still be strongly considered.

## Methodology Section

**Search Strategy:** 

Databases searched and dates covered:	CAB Abstracts (accessed via VetMed Resource) 1972–2022PubMed (accessed via pubmed.ncbi.nlm.nih.gov/) 1920–2022
Search strategy:	CAB Abstracts: gastric AND dilatation AND volvulus AND (dog OR canine) AND lactate gastric AND (dilatation OR dilation) AND (dog OR canine) AND lact* GDV AND lact* Gastric AND (dilatation OR dilation OR torsion) AND lact* PubMed: gastric AND dilatation AND volvulus AND (dog OR canine) AND lactate gastric AND (dilatation OR dilation) AND (dog OR canine) AND lact* GDV AND lact* Gastric AND (dilatation OR dilation OR torsion) AND lact*
Dates searches performed:	23 Feb 2022

**Exclusion / Inclusion Criteria:** 

Exclusion:	Dogs presenting for GDV where blood lactate measurement was not reported.
Inclusion:	All papers related to dogs presenting with GDV for which lactate measurements were taken, even if this was not the primary focus of the study.

**Search Outcome:** 

Database	Number of results	Excluded – Not relevant	Excluded – Review paper	Excluded – Not related to PICO question	Excluded – Duplicate paper	Total relevant papers
CAB Abstracts	31	4	4	7	1	15
PubMed	15	0	0	0	15	0
Total relevant papers						15

## ORCID

David Mackenzie: https://orcid.org/0000-0002-8751-4295

## Conflict of Interest

The author declares no conflicts of interest.

## Disclaimer

Knowledge Summaries are a peer-reviewed article type which aims to answer a clinical question based on the best available current evidence. It does not override the responsibility of the practitioner. Informed decisions should be made by considering such factors as individual clinical expertise and judgement along with patient’s circumstances and owners’ values. Knowledge Summaries are a resource to help inform and any opinions expressed within the Knowledge Summaries are the author's own and do not necessarily reflect the view of the RCVS Knowledge. Authors are responsible for the accuracy of the content. While the Editor and Publisher believe that all content herein are in accord with current recommendations and practice at the time of publication, they accept no legal responsibility for any errors or omissions, and make no warranty, express or implied, with respect to material contained within. For further information please refer to our Terms of Use.
